# Role of educational level in the relationship between Body Mass Index (BMI) and health-related quality of life (HRQL) among rural Spanish women

**DOI:** 10.1186/1471-2458-9-120

**Published:** 2009-04-30

**Authors:** María José García-Mendizábal, José Miguel Carrasco, Beatriz Pérez-Gómez, Nuria Aragonés, Pilar Guallar-Castillón, Fernando Rodríguez-Artalejo, Gonzalo López-Abente, Marina Pollán

**Affiliations:** 1 Department of Environmental Epidemiology and Cancer, National Centre for Epidemiology, Carlos III Institute of Health, Madrid, Spain; 2 Consortium for Biomedical Research in Epidemiology & Public Health (*CIBER en Epidemiología y Salud Pública *– *CIBERESP *-), Spain; 3 Aragon Health Sciences Institute, Zaragoza, Spain; 4 Department of Preventive Medicine and Public Health, School of Medicine, Universidad Autónoma de Madrid, Madrid, Spain

## Abstract

**Background:**

The impact of obesity on health-related quality of life (HRQL) has been little explored in rural areas. The goal of this study is to ascertain the association between obesity and HRQL among Spanish women living in a rural area, and the influence of their educational level.

**Methods:**

Cross-sectional study with personal interview of 1298 women (aged 18 to 60) randomly selected from the electoral rolls of 14 towns in Galicia, a region in the north-west of Spain. HRQL was assessed using the SF-36 questionnaire. The association between body mass index (BMI) and suboptimal scores in the different HRQL dimensions was summarised using odds ratios (ORs), obtained from multivariate logistic regression models. Separate analyses were conducted for women who had finished their education younger than 16 years old and women with secondary education to assess differences in the relationship between BMI and HRQL according to educational level.

**Results:**

Among women with primary or lower education, obesity was associated with a higher prevalence of suboptimal values in the following dimensions: Physical functioning (OR: 1.97; 95%CI: 1.22–3.18); Role-physical (OR: 1.81; 95%CI: 1.04–3.14); General health (OR: 1.76; 95%CI: 1.10–2.81); and Role-emotional (OR: 2.52; 95%CI: 1.27–5.03). In women with higher education, physical functioning was the only dimension associated with obesity (OR: 2.02: 95%CI 0.83–4.97).

**Conclusion:**

The impact of obesity on women's HRQL is greater among those with a lower educational level. This group registered higher prevalence of obesity and poorer self-perceived health.

## Background

Obesity is a multifactorial disorder stemming from the interaction between genetic and metabolic factors on the one hand, and nutritional lifestyles and physical activity on the other, both of which are, in turn, conditioned by social, behavioural and cultural factors. It constitutes a major and increasing public health problem worldwide [1]. In some industrialized countries such as USA [2] or Germany [3], the prevalence of obesity exceeds 25% in adults.

In Spain, the percentage of self-reported obesity among persons aged 20 years and over has increased in the last decades, rising from 7.7% in 1987 to 15.25% in 2006, in men and women alike [4,5]. Moreover, socio-economic level has been inversely related to self-reported obesity, particularly in women [6-9]. Although available data show that the prevalence of obesity rose from 1987 to 2003 across all groups of women, those with higher education registered the lowest increase, going from 3.9% in 1987 to 5.6% in 2003. This was in contrast to women without any formal education, among whom the prevalence of obesity, ascertained with reported measures, rose from 13.5% in 1987 to an alarming 27.8% in 2003 [5].

Obesity leads to a higher risk of hypertension, diabetes mellitus and cardiovascular disease [10-12]. Moreover, obesity also increases incidence of colon, breast, kidney, endometrium and oesophagus carcinomas[5,13,14]. Furthermore, excess weight has been associated with worse psychosocial well-being and quality of life [15,16], and a higher frequency of psychological disorders has been described in obese persons, probably related to a lower degree of social acceptance because of their physical appearance[17].

The relationship between obesity and HRQL has been widely investigated. Most general-population studies conclude that the quality of life of many obese persons displays suboptimal levels [18,19]. The association between obesity and HRQL is stronger in women than in men [20], in terms of both physical [16,18-24] and mental or psychosocial dimensions [15,24,25]. Educational level also has an influence on quality of life, inasmuch as the lower the educational level, the lower the quality-of-life score [26,27]. However, to date, no study in Spain has addressed the influence of educational level on the relationship between HRQL and obesity in the female population, using a generic HRQL measure such as the SF-36. This association can be of special interest in a rural setting, where educational levels, lifestyles and social relationships interact in a different way.

Accordingly, this study aims to examine the association between obesity and HRQL in women residing in a rural area of Galicia, a region in the north-west of Spain having one of the highest prevalences of obesity in the country [28] and whether this association varies in accordance with these women's educational level.

## Methods

From March to April 2004, we conducted a study to assess HRQL in the Galician population aged 18 to 60 years, residing in 7 coastal towns affected by the Prestige oil spill (which had occurred in November 2002) and in another 7 inland towns not affected by the oil spill but having similar socio-demographic characteristics. The study was part of a wider project which included both sexes, and its main characteristics have been published in detail elsewhere [29]. The coastal towns included in the study were Corcubión, Carnota, Fisterra, Laxe, Camariñas, Cée and Muxía. The inland area comprised the towns of Frades, Masía, Trazo, Tordoia, Cerceda, Oroso and Ordes. Study subjects were selected from municipal electoral rolls, using random sampling stratified by age, sex and town. According to previous experience of our group, municipal rolls often contain errors that make it impossible to contact a selected person. For this reason, three equivalent randomized samples of 2700 subject each (1373 men +1328 women) were selected. One of the three was considered the main sample, and each subject was assigned two substitutes with similar characteristics (sex, age and town), drawn from the other two samples. Hence, 1510 participants (56%) were drawn from the first list, 807 substitutes (30%) from the second list and 383 (14%) from the third. The main reasons for replacing the person of first choice were: flawed census data or impossibility of contact (32.7%); and refusal to respond (11.4%). For this study, of the total of 1328 women interviewed, we excluded 18 women who lacked BMI data and a further 12 interviews which provided no information on educational level. As a result, analyses were conducted on 1298 women.

Data were obtained from personal, home-based interviews conducted by trained interviewers. The questionnaire covered basic socio-demographic variables (sex, age, and educational level), work status, lifestyle (alcohol consumption, smoking, hours of sleep and sedentary leisure time), weight and height. HRQL was assessed by means of the SF-36 health questionnaire, a validated instrument [30] with normative information available for the Spanish population [31-33].

The SF-36 questionnaire measures 8 dimensions (Physical functioning, Role-physical, Bodily pain, General health, Vitality, Social functioning, Role-emotional and Mental health) on a scale ranging from 0 (worst score) to 100 (best score) [30]. The internal consistency indices (Cronbach's alpha) for each of the SF-36 questionnaire dimensions or scales were all above 0.7 in our data. Each dimension was investigated in two ways, by: (i) using the quantitative variable (scale of 0 to 100) and defining a dichotomous variable, suboptimal vs. optimal score; and, (ii) taking the same cut-off points as an earlier study [29]. In general, these cut-off points correspond to the 25^th ^percentile of the score distribution observed for each dimension in the overall population (men and women).

Both height and weight were self-reported by participants in the interviews. Body mass index (BMI) is the method of choice in both clinical and epidemiological practice for estimating the percentage of the obese population. BMI was calculated as follows: weight in kilograms divided by the square of the height in meters. Owing to the low number of women with BMI < 18.5 kg/m^2^, the following BMI categories were used: normal weight (< 25 kg/m^2^); overweight (≥ 25 to < 30 Kg/m^2^); and obesity (≥ 30 Kg/m^2^)[34].

Educational level was categorised by reference to the age at which each woman had completed her formal education, and whether or not a university degree had been obtained.

The Chi-square test was used to assess differences in the distribution of selected characteristics of the study population, according to BMI.

Comparison of mean values in HRQL scale scores by socio-demographic and life style variables was performed using univariate and bivariate linear regression models, with the latter including age due to its strong relationship with the dependent variable. In both cases, the statistical significance of these associations was tested by means of log-likelihood ratio tests.

Finally, two strategies were used to analyse the association between BMI and HRQL scale scores. Firstly, the statistical significance of the dose-response gradient was evaluated, by including BMI categories as a continuous variable in regression models that also included age, residential area, work status, education, smoking habit, daily number of hours of sleep and number of chronic diseases, alcohol consumption and sedentary leisure time (self-reported number of hours per day devoted to sedentary leisure activities, such as watching television or reading) as additional confounding factors. Secondly, the risk of having suboptimal values in each dimension was estimated by BMI category, using logistic regression models and taking the previously mentioned confounding variables into account.

To study the relationship between BMI and HRQL by educational level, separate analyses were also conducted for: women with primary education or lower ("No formal education" and "< 16 years"); and those with secondary and/or university education ("16–19 years", "> 19 years: no university education" and "University graduate"). A multiplicative interaction between BMI and educational level was tested, by including the corresponding interaction term in the overall model.

## Results

Additional file 1: Table S1 shows the characteristics of study participants by BMI category. Obesity was strongly associated with age and a lower educational level. The prevalence of obesity was higher in women with no sedentary leisure time, less than 7 hours' sleep and housewife or pensioner status, who were non-smokers and abstemious with regard to alcohol.

**Figure 1:**
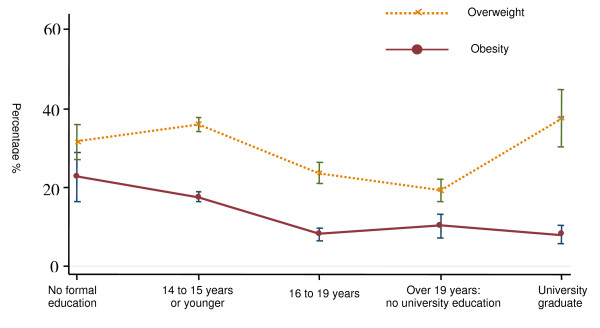
**Prevalence (95% confidence interval) of obesity according to educational level, adjusted for age**.

Figure 1 depicts the relationship between BMI and educational level. Overweight and obesity were more frequent in women with a lower educational level; indeed, prevalence of obesity in the two categories with a lower educational level exceeded 20%.

Set out in Table 1 are the mean scores obtained for each of the 8 dimensions of the SF-36 according to some explanatory variables. The age-adjusted statistical significance of the observed differences is shown. No differences in HRQL scores were observed between the two areas covered by this study, except in perceived mental health, which appeared to be worse among women from coastal towns. Some of the explanatory variables (work status, hours of sleep and sedentary leisure time) displayed statistically significant or marginally significant differences in all HRQL dimensions. However, alcohol consumption was not associated with HRQL when age was taken into account, and tobacco seemed to be exclusively linked to differences in mental health. Of special interest is the fact that the association of educational level with HRQL was only found with Physical functioning, General health, Vitality and Mental health SF-36 scales.

**Table 1:** Mean SF-36 scores for study participants: univariate analysis, by socio-demographic and lifestyle variables.

		**Physical Functioning**	**Role-Physical**	**Bodily Pain**	**General Health**	**Vitality**	**Social Functioning**	**Role-emotional**	**Mental Health**
		
	**n**								
									
**TOTAL POPULATION**	1298	91.6	88.5	81.0	66.7	65.6	91.5	93.9	73.7
									
**AGE**									
									
18–29 years	387	97.4	94.8	87.5	72.4	68.8	94.3	96.5	75.7
30–44 years	471	94.1	90.8	83.1	70.8	67.6	92.8	95.0	76.5
45–60 years	440	83.8	80.5	72.8	57.3	60.5	87.8	90.5	69.0
**p-value**		**< 0.001**	**< 0.001**	**< 0.001**	**< 0.001**	**< 0.001**	**< 0.001**	**< 0.001**	**< 0.001**
									
**RESIDENTIAL AREA**									
									
Inland towns	652	91.1	88.5	80.0	66.5	65.2	92.0	94.2	75.1
Coastal towns	646	92.1	88.5	81.9	66.9	65.9	91.1	93.5	72.3
**Age-adjusted p-value**		0.150	0.919	0.130	0.551	0.501	0.403	0.571	0.013
									
**EDUCATIONAL LEVEL**^a^									
									
No formal education	138	81.8	80.1	74.0	52.4	56.5	86.6	87.2	64.5
< 16 years	588	89.4	86.3	78.1	64.5	64.9	90.9	93.4	73.6
16–19 years	295	95.6	93.3	85.6	71.8	68.5	93.7	96.5	75.8
> 19 yrs: no university education	160	96.8	92.5	86.0	72.1	68.3	93.4	95.8	76.3
University graduate	117	96.6	91.7	84.9	74.2	68.4	92.3	94.6	76.3
**Age-adjusted p-value**		**< 0.001**	0.775	0.733	**< 0.001**	**0.008**	0.595	0.112	**0.002**
									
**WORK STATUS**									
									
Employed	591	93.6	91.0	81.5	69.7	65.7	92.8	95.7	74.5
Unemployed	101	93.7	89.4	79.4	68.1	68.4	90.2	95.1	74.1
Pre-job market ^b^	159	97.3	94.8	89.8	74.0	71.3	94.7	96.2	78.4
Pensioner	50	70.4	73.0	64.7	45.3	52.9	74.0	86.0	60.8
Housewife	390	88.2	83.6	78.7	61.4	64.0	90.9	90.9	72.4
**Age-adjusted p-value**		**< 0.001**	**0.018**	**0.008**	**< 0.001**	**0.003**	**< 0.001**	**0.023**	**0.001**
									
**HOURS OF SLEEP**									
									
< 7 hours	181	85.3	79.8	69.8	57.6	56.7	85.0	89.9	64.2
7 – 9 hours	901	92.9	90.2	83.3	68.1	66.9	93.0	95.3	75.3
> 9 hours	216	91.5	88.7	80.8	68.3	67.6	91.0	91.4	75.2
**Age-adjusted p-value**		**< 0.001**	**0.004**	**< 0.001**	**< 0.001**	**< 0.001**	**< 0.001**	**0.003**	**< 0.001**
									
**SEDENTARY LEISURE TIME **^c^									
									
None	60	80.2	77.9	68.6	54.2	53.6	82.7	85.6	62.3
< 3 hours	791	92.2	88.9	81.1	67.3	66.2	92.2	94.4	74.4
3 – 5 hours	414	92.00	89.6	82.6	67.2	66.0	91.6	93.9	74.0
> 5 hours	31	92.1	82.3	77.3	69.7	68.5	92.3	96.8	74.2
**Age-adjusted p-value**		**< 0.001**	0.090	**0.016**	**0.006**	**0.001**	**0.012**	0.062	**0.001**
									
**TOBACCO SMOKING**									
									
Non-smoker	906	90.35	87.5	80.4	65.2	64.9	91.5	93.9	73.7
Ex-smoker	69	92.4	88.4	79.4	71.2	67.3	92.6	92.8	74.3
0–10 cig/day	133	95.6	90.6	83.9	70.3	67.2	91.2	95.5	74.4
10–19 cig/day	103	95.6	94.2	84.0	70.2	68.7	91.5	93.2	76.1
> 20 cig/day	82	93.7	87.8	79.0	68.7	64.9	91.0	92.3	68.7
**Age-adjusted p-value**		0.087	0.688	0.683	0.303	0.745	0.802	0.723	0.063
									
**ALCOHOL CONSUMPTION**									
									
Abstemious		91.0	87.5	81.0	65.9	65.8	91.5	93.5	73.8
Moderate		94.1	91.5	82.0	69.6	64.6	92.7	94.4	74.2
excessive		92.8	91.1	79.4	68.1	65.0	90.4	95.4	72.8
**Age-adjusted p-value**		0.891	0.624	0.224	0.819	0.154	0.495	0.716	0.570

^a ^Age at which education completed.

^b ^Women studying or seeking first job.

^c ^Self-reported number of hours per day devoted to sedentary leisure activities, such as watching television or reading.

**Figure 2:**
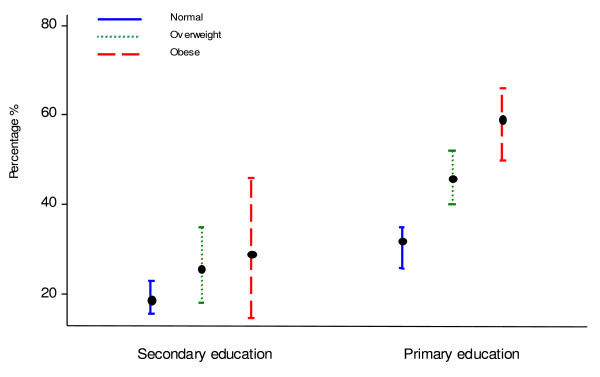
**Prevalence (95% confidence interval) of women with suboptimal values in general health, by BMI category and education**.

Figure 2 show the prevalences of women with suboptimal values in General health, by educational level and BMI. A noteworthy finding was the increasing percentage of women reporting suboptimal HRQL values among overweight and obese women in the primary education group.

Table 2 presents the association between obesity and HRQL, overall and by educational level. The scores in physical functioning, role-physical, bodily pain, general health and vitality decreased with BMI (p for linear trend < 0.05 in all cases). This translates as a higher frequency of suboptimal scores among women having a higher BMI, with statistically significant ORs in the dimensions of Physical functioning (overweight OR: 1.75; obesity OR: 1.96), Role-physical (obesity OR: 1.62), General health (overweight OR: 1.35; obesity OR: 1.65) and Role-emotional (obesity OR: 1.89).

**Table 2:** Mean SF-36 scores by BMI category, and multivariate association between SF-36 sub-optimal scores and obesity.

	Overall analysis (n = 1298)					Stratified analysis									
						Primary education^b^					Secondary education^c^				
	Mean score		Suboptimal values			Mean score		Suboptimal values			Mean score		Suboptimal values		
		p-value^a^	n (%)	OR^3^	95% CI		p-value^a^	n (%)	OR^3^	95% CI		p-value^a^	n (%)	OR^3^	95% CI
**SF-36 BMI Physical functioning**															
Normal	95.3		121 (17)	1.00		93.0		67 (25)	1.00		96.7		54 (12)	1.00	
Overweight	89.3		155 (38)	**1.75**	1.26 – 2.42	87.2		134 (44)	**1.88**	1.26 – 2.82	95.5		21 (20)	1.40	0.75 – 2.62
Obese	83.0	**0.001**	97 (51)	**1.96**	1.30 – 2.96	81.1	**0.005**	85 (54)	**1.97**	1.22 – 3.18	91.7	0.508	12 (34)	2.02	0.83 – 4.97
**Role-physical**															
Normal	92.5		77 (11)	1.00		91.3		35 (13)	1.00		93.3		42 (10)	1.00	
Overweight	86.9		72 (18)	1.12	0.75 – 1.67	84.8		60 (20)	1.27	0.77 – 2.09	92.8		12 (12)	0.78	0.36 – 1.67
Obese	77.1	**0.014**	55 (29)	**1.62**	1.02 – 2.58	75.2	**0.019**	49 (31)	**1.81**	1.04 – 3.14	85.7	0.954	6 (17)	1.16	0.40 – 3.37
**Bodily pain**															
Normal	84.9		278 (49)	1.00		82.7		121 (45)	1.00		86.3		157 (36)	1.00	
Overweight	79.2		200 (49)	1.04	0.78 – 1.37	77.4		157 (52)	1.03	0.72 – 1.48	84.5		43 (41)	0.99	0.62 – 1.60
Obese	70.3	**0.011**	115 (60)	1.29	0.89 – 1.88	68.1	**0.015**	99 (63)	1.36	0.86 – 2.13	80.0	0.973	16 (46)	1.10	0.51 – 2.36
**General health**															
Normal	71.1		167 (24)	1.00		67.6		84 (32)	1.00		73.3		83 (19)	1.00	
Overweight	63.0		166 (41)	**1.35**	0.98 – 1.84	60.8		139 (46)	1.38	0.93 – 2.03	69.6		27 (26)	1.25	0.71 – 2.21
Obese	58.2	**0.030**	102 (53)	**1.65**	1.11 – 2.46	55.8	0.083	92 (59)	**1.76**	1.10 – 2.81	69.1	0.796	10 (29)	0.96	0.38 – 2.43
**Vitality**															
Normal	68.1		94 (13)	1.00		66.8		43 (16)	1.00		68.9		51 (12)	1.00	
Overweight	64.7		81 (20)	1.16	0.79 – 1.70	63.7		68 (22)	1.24	0.77 – 2.01	67.8		13 (13)	0.97	0.46 – 2.04
Obese	58.2	**0.043**	57 (30)	1.45	0.91 – 2.29	56.7	**0.038**	50 (32)	1.51	0.88 – 2.60	64.9	0.732	7 (20)	1.48	0.54 – 4.02
**Social functioning**															
Normal	92.9		151 (22)	1.00		91.6		59 (22)	1.00		93.7		92 (21)	1.00	
Overweight	90.5		113 (28)	1.11	0.80 – 1.55	90.0		83 (27)	1.10	0.72 – 1.69	91.9		30 (29)	1.24	0.73 – 2.14
Obese	88.7	0.464	68 (35)	1.27	0.84 – 1.92	87.8	0.512	59 (38)	1.40	0.86 – 2.29	92.9	0.723	9 (26)	0.84	0.34 – 2.05
**Role-emotional**															
Normal	96.0		46 (7)	1.00		95.9		19 (7)	1.00		96.0		27 (6)	1.00	
Overweight	93.0		46 (11)	1.35	0.83 – 2.23	92.2		36 (12)	1.53	0.80 – 2.91	95.2		10 (10)	1.03	0.42 – 2.51
Obese	88.2	0.172	35 (18)	**1.89**	1.07 – 3.35	86.2	**0.039**	32 (20)	**2.52**	1.27 – 5.03	97.1	0.259	3 (9)	0.82	0.19 – 3.51
**Mental health**															
Normal	75.0		195 (28)	1.00		73.2		74 (28)	1.00		76.0		121 (28)	1.00	
Overweight	73.1		135 (33)	1.04	0.76 – 1.42	72.1		102 (34)	1.09	0.72 – 1.65	75.8		33 (32)	1.06	0.63 – 1.77
Obese	70.5	0.176	75 (39)	0.99	0.66 – 1.48	69.2	0.355	64 (41)	1.10	0.68 – 1.80	76.7	0.299	11 (31)	0.84	0.36 – 1.93

General results and stratified analysis by educational group.

^a^Test for trend adjusted for age, residential area, work status, education, smoking habit, daily number of hours of sleep and number of chronic diseases, alcohol consumption, sedentary leisure time

^b^n Normal (**BMI < 25 Kg/m^3^) **= 266; overweight **(BMI ≥ 25 Kg/m^3 ^< 30 Kg/m) **= 303; obese (**BMI ≥ 30 Kg/m^3^) **= 157

^c^n Normal (**BMI < 25 Kg/m^3^) **= 433; overweight **(BMI ≥ 25 Kg/m^3 ^< 30 Kg/m) **= 104; obese (**BMI ≥ 30 Kg/m^3^) **= 35

OR adjusted for age, area, work status, education, smoking habit, daily number of hours of sleep and number of chronic diseases, alcohol consumption, sedentary leisure time

Risk estimators for the covariates included in these models are provided by way of supplementary information (Additional file 2). Age was positively associated with suboptimal values of Physical functioning, Role-physical and Bodily pain, while intermediate ages seemed to register better Mental health scores. Pensioners and housewives reported lower HRQL results, mainly on the Role-physical, General health and Role-emotional scales. Pensioners also displayed a high prevalence of suboptimal values in Social functioning and Mental health. Women who slept for less than 7 hours had a greater prevalence of suboptimal values in Bodily pain, Vitality, Social functioning and Mental health. Whereas reporting any sedentary leisure time implied a lower frequency of suboptimal values in Mental health, heavy smokers were more prone to low scores in this dimension.

Table 2 also summarises the results yielded by our analysis of the relationship between HRQL and BMI, stratified by educational level. Among women with primary education or lower, statistically significant differences in SF-36 scores were observed, with a downward trend in accordance with BMI in all SF-36 dimensions except Social functioning, Mental health and General health. In this group, multivariate analysis showed that women with a greater BMI registered a higher frequency of suboptimal values, with ORs equal to or higher than 1.50 for several physical health dimensions: Physical functioning (overweight OR: 1.88; 95% CI: 1.26–2.82; obesity OR: 1.97; 95% CI: 1.22–3.18), Role-physical (obesity OR: 1.81; 95% CI: 1.04–3.14) and General health (obesity OR: 1.76; 95% CI: 1.10–2.81), as well as in two mental health dimensions: Vitality (obesity OR: 1.51; 95% CI: 0.88–2.60) and Role-emotional (obesity OR: 2.52; 95% CI: 1.27–5.03).

Our results show a less marked association between BMI and HRQL in more educated women. Women with secondary education presented differences in mean scores with BMI in some scales, but adjusted trends were not significant in any case. In the multivariate logistic regression analysis, Physical functioning was the only dimension with higher prevalence of suboptimal scores among obese women, even though it did not attained statistical significance (OR: 2.02; 95%CI 0.83–4.97). However, the prevalence of obesity among more educated women was rather low and the study sample was of insufficient size to demonstrate an interaction between educational level and BMI.

## Discussion

This study highlights the way in which BMI and educational level are directly linked to HRQL, particularly as regards physical health dimensions. In general, scores on all SF-36 scales decreased as BMI rose and educational level fell.

This study took advantage of a relatively large sample of women that had been randomly recruited in two rural areas of Galicia, and although selection bias is within the bounds of possibility, the sample design (three equivalent samples) reduce this possibility. Furthermore, the proportion of refusals to respond was reasonably low, i.e., 11.4%. The study aimed to provide information on HRQL in rural areas affected and unaffected by the Prestige oil spill, one and a half years after the accident, yet no differences in HRQL were observed between these two areas. It should be noted here that the study's cross-sectional design limits any causal inference.

One possible limitation of our study is that both height and weight were self-reported. According to published studies, self-report measures generally underestimate obesity. Moreover, this problem is said to increase with age and measured obesity [35]. The same results have been observed for Spain [36]. However, the extent to which this bias varies with socio-economic/educational level is less clear. Several studies describe higher underreporting of BMI among women with less qualified occupations but the differences fail to attain statistical significance [37]. In terms of educational level, some studies have also found an increase in underreporting with educational level among subjects in general [38], and among women in particular [39]. Our study assesses the relationship between HRQL and obesity among women, and concludes that there are no significant differences in any of SF-36 scales as between normal, overweight and obese females in the better-educated category. If the above-mentioned bias were present in our data, a number of obese women could be misclassified as overweight. Since mean SF-36 scale scores among overweight women are in all cases higher than scores in the obese group, real differences among groups might thus be even smaller than those observed. Another possible limitation is that, in spite of the multivariate analysis, we can not exclude the influence of other residual confusion factors that may be linked to HRQL, nor rule out the possibility that some of our results could be due to chance. Regarding statistical analysis, a lot of comparisons have been carried out, since we have evaluated the relationship between obesity and each of SF-36 scales, overall and by educational group. In this context, some of our statistical significant associations may be just chance findings. For this reason, we took into account not only statistical significance, but the strength of the association as well as mean differences in score. Statistical power was not enough to allow the use of alternative methods to correct for multiple testing.

A further relevant point warranting mention is the fact that the prevalence of obesity was substantially lower among women with secondary education (6% versus 22%), something in agreement with other studies [5-9]. Thus, the low number of obese women in the secondary-education group implies low statistical power to detect an interaction between education and BMI. In fact, interaction terms were not statistically significant, even though effect estimators seemed to be substantially different in the two educational groups.

As expected, age and BMI exhibited the strongest association with HRQL. Our results show that obesity has a greater influence on HRQL than other factors studied, such as smoking, alcohol consumption, sedentary lifestyle and work status. Tobacco use was solely related to mental health, in line with previous reports [40]. Work status and hours of sleep were also associated with HRQL and should be considered as possible confounders. The negative association between number of hours of sleep and several mental dimensions might reflect the influence of sleeping disturbances, such as insomnia [41]. Some syndromes like obstructive sleep apnea are widely prevalent in patients with obesity and could also affect sleep; however we could not deepen into these aspects as our questionnaire did not include information on this topic.

The frequency of obese persons is higher in rural areas or in towns of less than 100,000 inhabitants compared to rates observed for large cities [28]. The prevalence of obesity in our population (14.53%) was slightly higher than that reported by the 2003 National Health Survey for the female population (13.6%). Moreover, our results show an association between overweight/obesity and educational level in women, which has already been previously described in the literature. In Spain, cross-sectional studies undertaken from 1990 to 2000 on random samples representative of the populations of the regions of Andalusia, the Balearic Isles, the Canary Islands, Catalonia, Galicia, Madrid, the Basque Country and the Valencian Region, reported a higher prevalence of obesity in subgroups -male and female- having a lower educational level. Other studies, both in Spain [8] and in other developed countries [7,9], have described an inverse relationship between socio-economic level and BMI. The greater prevalence of obesity among the population with a lower education has been attributed to certain health behaviours, such as fat-rich diets or the low frequency of regular physical exercise [42].

Our results also show that, among Galician women living in rural areas, overweight and obesity was associated with worse HRQL, particularly in the physical health dimensions. A similar result has been reported in a multicentric study aimed to design a HRQL questionnaire in post-menopausal Spanish women [43,44]. The analysis of the Spanish National Health Survey made by Guallar et al. [20] also showed a significant positive dose-response relationship of BMI with self-rated health status and utilisation of health-care services in women, which did not vary with age, educational level or presence of chronic disease, though they did not study this association by HRQL dimensions, as the Survey did not include specific questionnaires on this issue.

Our results show that the afore mentioned association is stronger in women with a lower educational level, since obese women with primary education invariably register lower scores (up to 11 points on the Role-emotional scale) than do those with secondary education. The fact that obese women with primary education have a greater risk of scoring suboptimal values in the Role-physical, General health and Role-emotional dimensions, compared to women with secondary education may give an idea of the different subjective perceptions of health held by these two groups [45]. Whereas physical functioning evaluates the degree to which health limits physical activities, such as self-care, walking and the like, Role-physical and Role-emotional ascertain how such limitations (physical or emotional respectively) interfere with work and other activities of daily living, including subjectively substandard performance [32,46]. This difference in results could be due to a different perception of limitations, related to educational level. Physical functioning is in general limited by obesity, yet women with lower education perceive their work performance as being worse. It is to be expected that differences in daily activities performed by one or another group of women in accordance with their educational level, would explain these results; probably in this population obesity affects more to women who largely perform tasks which entail more physical strain in both physical and mental health dimensions, as reflect our results in Role-physical and Role-emotional scales.

The impact of obesity on mental health is controversial: whilst some studies conclude that obesity cannot be associated with an increase in psychopathologies [47], others report worse HRQL scores for obese women in the mental dimensions [24], though these contradictory results could be due to differences in psychological well-being, one of the strongest correlate of HRQL components [48]. In our study, obese subjects did not report anxiety more frequently than did the rest of the women, and we failed to show worse Mental health scores in this group. However, when this association was analysed separately by educational level, obese women with primary education or lower registered a mean SF-36 Mental health score which was more than 3 points lower than that of women with a normal BMI, whereas scarcely any BMI-related differences were observed in the mental health reported by women with secondary education.

Obesity also involves social problems, manifested as discrimination and lower social mobility. Gortmarker and colleagues observed fewer marriages, lower income and a lower educational level among persons suffering from obesity during adolescence [49]. Unfortunately, we don't have information about individual participant incomes, which could be a modifying factor in the relationship among educative level, obesity and HRQL. Since the effect of social mobility, construed as a decline in social level with respect to that of birth, is greater among women than among men [8] obesity might accentuate gender inequalities in this respect [50]. From a public health point of view, the reduction in BMI among women with a higher socio-economic level, detected by the most recent health surveys [5], lends support to the interest attached to targeting a specific message regarding obesity prevention at the most disadvantaged socio-economic groups.

## Conclusion

Health interventions targeted at fighting obesity must consider the higher prevalence of obese women in population segments with a lower educational level, in whom overweight-associated limitations seem to have greater repercussion in both physical and emotional roles. The different effect of obesity in HRQL observed among women with higher and lower education also suggests that education may attenuate the negative impact of obesity on women's health-related quality of life.

## Abbreviations

HRQL: health-related quality of life; BMI: Body Mass Index; OR: odds ratio; MHC: Ministry of Health & Consumer Affairs; WHO: World Health Organisation.

## Competing interests

The authors declare that they have no competing interests.

## Authors' contributions

MJGM and JMC conceived the idea, carried out the statistical analysis and wrote the manuscript. BPG, NA, PGC, FRA and GLA made contribution to statistical analyses and interpretation of results, and revised the manuscript for important intellectual content. MP designed the study, contributed to manuscript writing, and revised it for important intellectual content. All authors contributed to the final version of the manuscript.

## Pre-publication history

The pre-publication history for this paper can be accessed here:



## Supplementary Material

Additional file 1 **BMI by study participants' characteristics** Click here for file

Additional file 2 **Supplementary information**. Effect estimators for covariates included in the multivariate logistic regression models. Click here for file
