# Supplementary material for: Role of educational level in the relationship between Body Mass Index (BMI) and health-related quality of life (HRQL) among rural Spanish women
Source: BMC Public Health. 2009 Apr 30;9:120. doi: 10.1186/1471-2458-9-120 (PMC2696428; doi:10.1186/1471-2458-9-120)
Supplement: Additional file 1 — BMI by study participants' characteristics [file 1471-2458-9-120-S1.doc]

Additional file 1

Table S1: Distribution of BMI by study participants' characteristics
