# Supplementary material for: Role of educational level in the relationship between Body Mass Index (BMI) and health-related quality of life (HRQL) among rural Spanish women
Source: BMC Public Health. 2009 Apr 30;9:120. doi: 10.1186/1471-2458-9-120 (PMC2696428; doi:10.1186/1471-2458-9-120)
Supplement: Additional file 2 — Supplementary information. Effect estimators for covariates included in the multivariate logistic regression models. [file 1471-2458-9-120-S2.doc]

### Supplementary information. Effect estimators for covariates included in the multivariate logistic regression models.

|  |  |  | Physical functioning | | | | | **Role-physical** | | | | | Bodily pain | | | | | **General health** | | | | | **Vitality** | | | | | **Social functioning** | | | | | **Role-emotional** | | | | | **Mental health** | | | | |
| --- | --- | --- | --- | --- | --- | --- | --- | --- | --- | --- | --- | --- | --- | --- | --- | --- | --- | --- | --- | --- | --- | --- | --- | --- | --- | --- | --- | --- | --- | --- | --- | --- | --- | --- | --- | --- | --- | --- | --- | --- | --- | --- |
|  |  |  | Suboptimal values | | | | | Suboptimal values | | | | | Suboptimal values | | | | | Suboptimal values | | | | | Suboptimal values | | | | | Suboptimal values | | | | | Suboptimal values | | | | | Suboptimal values | | | | |
|  |  | N | n | OR | 95%CI | | | n | OR | 95%CI | | | n | OR | 95%CI | | | n | OR | 95%CI | | | n | OR | 95%CI | | | n | OR | 95%CI | | | n | OR | 95%CI | | | n | OR | 95%CI | | |
|  | Age |  |  |  |  |  |  |  |  |  |  |  |  |  |  |  |  |  |  |  |  |  |  |  |  |  |  |  |  |  |  |  |  |  |  |  |  |  |  |  |  |  |
|  | 18-29 years | 387 | 44 | 1.00 |  |  |  | 32 | 1.00 |  |  |  | 131 | 1.00 |  |  |  | 86 | 1.00 |  |  |  | 49 | 1.00 |  |  |  | 79 | 1.00 |  |  |  | 25 | 1.00 |  |  |  | 110 | 1.00 |  |  |  |
|  | 30-44 years | 471 | 115 | **1.87** | 1.14 | - | 3.06 | 60 | 1.45 | 0.82 | - | 2.58 | 199 | 1.23 | 0.86 | - | 1.75 | 118 | **0.64** | 0.42 | - | 0.96 | 70 | 0.77 | 0.47 | - | 1.25 | 109 | 1.06 | 0.70 | - | 1.61 | 39 | 1.11 | 0.57 | - | 2.19 | 125 | **0.64** | 0.44 | - | 0.94 |
|  | 45-60 years | 440 | 214 | **2.79** | 1.65 | - | 4.73 | 112 | **2.19** | 1.18 | - | 4.06 | 263 | **1.95** | 1.30 | - | 2.92 | 231 | 1.08 | 0.69 | - | 1.69 | 113 | 0.80 | 0.47 | - | 1.38 | 144 | 1.20 | 0.75 | - | 1.92 | 63 | 1.23 | 0.59 | - | 2.58 | 170 | 0.74 | 0.48 | - | 1.15 |
|  | Residential area |  |  |  |  |  |  |  |  |  |  |  |  |  |  |  |  |  |  |  |  |  |  |  |  |  |  |  |  |  |  |  |  |  |  |  |  |  |  |  |  |  |
|  | Inland towns | 652 | 210 | 1.00 |  |  |  | 101 | 1.00 |  |  |  | 311 | 1.00 |  |  |  | 216 | 1.00 |  |  |  | 122 | 1.00 |  |  |  | 153 | 1.00 |  |  |  | 59 | 1.00 |  |  |  | 184 | 1.00 |  |  |  |
|  | Coastal towns | 646 | 163 | **0.61** | 0.46 | - | 0.82 | 103 | 1.02 | 0.73 | - | 1.43 | 282 | 0.79 | 0.62 | - | 1.00 | 219 | 0.95 | 0.72 | - | 1.24 | 110 | 0.77 | 0.55 | - | 1.07 | 179 | 1.21 | 0.91 | - | 1.59 | 68 | 1.22 | 0.80 | - | 1.85 | 221 | 1.21 | 0.93 | - | 1.58 |
|  | Work status |  |  |  |  |  |  |  |  |  |  |  |  |  |  |  |  |  |  |  |  |  |  |  |  |  |  |  |  |  |  |  |  |  |  |  |  |  |  |  |  |  |
|  | Employed | 591 | 147 | 1.00 |  |  |  | 73 | 1.00 |  |  |  | 267 | 1.00 |  |  |  | 161 | 1.00 |  |  |  | 101 | 1.00 |  |  |  | 135 | 1.00 |  |  |  | 41 | 1.00 |  |  |  | 176 | 1.00 |  |  |  |
|  | Unemployed | 101 | 21 | 0.85 | 0.47 | - | 1.52 | 13 | 1.17 | 0.59 | - | 2.31 | 49 | 1.30 | 0.83 | - | 2.05 | 32 | 1.33 | 0.80 | - | 2.20 | 20 | 1.15 | 0.64 | - | 2.08 | 29 | 1.26 | 0.75 | - | 2.11 | 8 | 1.06 | 0.45 | - | 2.50 | 33 | 1.12 | 0.68 | - | 1.84 |
|  | Pre-job market | 159 | 18 | 1.13 | 0.58 | - | 2.21 | 14 | 1.36 | 0.64 | - | 2.89 | 46 | 0.72 | 0.45 | - | 1.14 | 28 | 0.96 | 0.56 | - | 1.66 | 11 | 0.45 | 0.21 | - | 0.95 | 29 | 0.90 | 0.52 | - | 1.55 | 11 | 1.66 | 0.69 | - | 3.97 | 33 | 0.65 | 0.38 | - | 1.08 |
|  | Pensioner | 50 | 33 | 2.05 | 1.00 | - | 4.21 | 19 | **2.07** | 1.01 | - | 4.23 | 33 | 1.21 | 0.62 | - | 2.38 | 36 | **2.81** | 1.37 | - | 5.79 | 20 | 1.28 | 0.61 | - | 2.66 | 25 | **2.05** | 1.04 | - | 4.02 | 11 | 1.70 | 0.72 | - | 4.04 | 29 | **2.11** | 1.07 | - | 4.16 |
|  | Housewife | 390 | 153 | 1.22 | 0.88 | - | 1.70 | 85 | **1.54** | 1.04 | - | 2.28 | 196 | 0.99 | 0.74 | - | 1.33 | 176 | **1.56** | 1.13 | - | 2.14 | 78 | 0.89 | 0.60 | - | 1.31 | 113 | 1.16 | 0.83 | - | 1.63 | 55 | **1.81** | 1.10 | - | 2.97 | 131 | 1.13 | 0.82 | - | 1.55 |
|  | Hours of sleep |  |  |  |  |  |  |  |  |  |  |  |  |  |  |  |  |  |  |  |  |  |  |  |  |  |  |  |  |  |  |  |  |  |  |  |  |  |  |  |  |  |
|  | <7 hours | 181 | 79 | 1.36 | 0.92 | - | 2.00 | 48 | 1.51 | 0.99 | - | 2.30 | 116 | **1.80** | 1.27 | - | 2.57 | 88 | 1.36 | 0.94 | - | 1.99 | 58 | **1.74** | 1.15 | - | 2.62 | 73 | **1.71** | 1.18 | - | 2.46 | 28 | 1.37 | 0.81 | - | 2.30 | 97 | **2.37** | 1.66 | - | 3.37 |
|  | 7 - 9 hours | 901 | 234 | 1.00 |  |  |  | 125 | 1.00 |  |  |  | 381 | 1.00 |  |  |  | 281 | 1.00 |  |  |  | 142 | 1.00 |  |  |  | 203 | 1.00 |  |  |  | 74 | 1.00 |  |  |  | 249 | 1.00 |  |  |  |
|  | > 9 hours | 216 | 60 | 0.95 | 0.64 | - | 1.42 | 31 | 0.96 | 0.60 | - | 1.54 | 96 | 1.08 | 0.78 | - | 1.50 | 66 | 0.79 | 0.54 | - | 1.15 | 32 | 0.83 | 0.52 | - | 1.31 | 56 | 1.18 | 0.81 | - | 1.72 | 25 | 1.43 | 0.84 | - | 2.44 | 59 | 0.94 | 0.65 | - | 1.36 |
|  | Sedentary leisure time |  |  |  |  |  |  |  |  |  |  |  |  |  |  |  |  |  |  |  |  |  |  |  |  |  |  |  |  |  |  |  |  |  |  |  |  |  |  |  |  |  |
|  | None | 60 | 29 | 1.00 |  |  |  | 16 | 1.00 |  |  |  | 38 | 1.00 |  |  |  | 31 | 1.00 |  |  |  | 22 | 1.00 |  |  |  | 21 | 1.00 |  |  |  | 10 | 1.00 |  |  |  | 34 | 1.00 |  |  |  |
|  | < 3 hours | 791 | 221 | 0.60 | 0.32 | - | 1.11 | 125 | 0.79 | 0.40 | - | 1.54 | 354 | 0.62 | 0.35 | - | 1.10 | 262 | 0.73 | 0.40 | - | 1.34 | 127 | 0.43 | 0.23 | - | 0.81 | 193 | 0.83 | 0.45 | - | 1.53 | 76 | 0.83 | 0.37 | - | 1.88 | 237 | **0.42** | 0.23 | - | 0.75 |
|  | 3-5 hours | 414 | 112 | 0.71 | 0.37 | - | 1.36 | 57 | 0.63 | 0.31 | - | 1.29 | 186 | 0.71 | 0.39 | - | 1.30 | 131 | 0.67 | 0.35 | - | 1.26 | 78 | 0.61 | 0.31 | - | 1.17 | 107 | 0.84 | 0.44 | - | 1.58 | 40 | 0.70 | 0.30 | - | 1.65 | 123 | **0.40** | 0.22 | - | 0.73 |
|  | > 5 hours | 31 | 10 | 1.27 | 0.41 | - | 3.92 | 6 | 1.02 | 0.30 | - | 3.48 | 14 | 0.82 | 0.31 | - | 2.18 | 9 | 0.64 | 0.21 | - | 1.97 | 5 | 0.52 | 0.15 | - | 1.87 | 11 | 1.52 | 0.55 | - | 4.19 | 1 | 0.16 | 0.17 | - | 1.52 | 11 | 0.57 | 0.21 | - | 1.55 |
|  | Tobacco smoking |  |  |  |  |  |  |  |  |  |  |  |  |  |  |  |  |  |  |  |  |  |  |  |  |  |  |  |  |  |  |  |  |  |  |  |  |  |  |  |  |  |
|  | Non-smoker | 906 | 296 | 1.00 |  |  |  | 153 | 1.00 |  |  |  | 419 | 1.00 |  |  |  | 329 | 1.00 |  |  |  | 170 | 1.00 |  |  |  | 225 | 1.00 |  |  |  | 85 | 1.00 |  |  |  | 279 | 1.00 |  |  |  |
|  | Ex-smoker | 69 | 17 | 0.80 | 0.42 | - | 1.51 | 10 | 0.89 | 0.42 | - | 1.89 | 36 | 1.35 | 0.80 | - | 2.30 | 19 | 0.91 | 0.49 | - | 1.69 | 8 | 0.61 | 0.27 | - | 1.39 | 21 | 1.28 | 0.71 | - | 2.29 | 8 | 1.30 | 0.55 | - | 3.07 | 21 | 0.93 | 0.52 | - | 1.69 |
|  | 0-10 cig/day | 133 | 19 | 0.51 | 0.29 | - | 0.90 | 17 | 1.07 | 0.60 | - | 1.90 | 54 | 0.98 | 0.66 | - | 1.46 | 38 | 1.02 | 0.65 | - | 1.60 | 22 | 1.06 | 0.61 | - | 1.84 | 34 | 1.28 | 0.82 | - | 2.02 | 12 | 1.39 | 0.70 | - | 2.78 | 37 | 0.97 | 0.62 | - | 1.51 |
|  | 10-19 cig/day | 103 | 19 | 0.76 | 0.43 | - | 1.36 | 11 | 0.86 | 0.43 | - | 1.71 | 43 | 0.97 | 0.63 | - | 1.51 | 25 | 0.83 | 0.50 | - | 1.39 | 15 | 0.91 | 0.49 | - | 1.70 | 26 | 1.19 | 0.72 | - | 1.96 | 11 | 1.82 | 0.89 | - | 3.76 | 30 | 0.98 | 0.60 | - | 1.59 |
|  | >20 cig/day | 82 | 21 | 0.83 | 0.46 | - | 1.49 | 13 | 1.04 | 0.53 | - | 2.03 | 40 | 1.09 | 0.67 | - | 1.78 | 23 | 0.74 | 0.42 | - | 1.30 | 17 | 1.09 | 0.58 | - | 2.07 | 25 | 1.21 | 0.70 | - | 2.08 | 11 | 1.86 | 0.88 | - | 3.94 | 37 | **1.77** | 1.07 | - | 2.92 |
|  | Alcohol consumption |  |  |  |  |  |  |  |  |  |  |  |  |  |  |  |  |  |  |  |  |  |  |  |  |  |  |  |  |  |  |  |  |  |  |  |  |  |  |  |  |  |
|  | Abstemious | 965 | 298 | 1.00 |  |  |  | 157 | 1.00 |  |  |  | 431 | 1.00 |  |  |  | 345 | 1.00 |  |  |  | 175 | 1.00 |  |  |  | 248 | 1.00 |  |  |  | 96 | 1.00 |  |  |  | 302 | 1.00 |  |  |  |
|  | Moderate | 167 | 39 | 1.12 | 0.70 | - | 1.77 | 22 | 1.15 | 0.68 | - | 1.94 | 75 | 1.35 | 0.94 | - | 1.94 | 41 | 0.78 | 0.51 | - | 1.21 | 27 | 1.14 | 0.69 | - | 1.88 | 36 | 0.91 | 0.59 | - | 1.40 | 16 | 1.19 | 0.64 | - | 2.21 | 49 | 1.05 | 0.71 | - | 1.57 |
|  | Excessive | 166 | 46 | 1.27 | 0.83 | - | 1.94 | 25 | 1.13 | 0.69 | - | 1.86 | 87 | **1.66** | 1.16 | - | 2.37 | 49 | 0.91 | 0.69 | - | 1.36 | 30 | 1.24 | 0.77 | - | 2.00 | 48 | 1.34 | 0.90 | - | 2.00 | 15 | 0.97 | 0.52 | - | 1.82 | 54 | 1.14 | 0.77 | - | 1.68 |

Odds Ratios obtained from multivariate logistic models that included the following variables: age; residential area; work status; education; smoking habit; daily number of hours of sleep and number of chronic diseases; body mass index; alcohol consumption.
